# Supplementary material for: Prevalence of burnout among university students in low- and middle-income countries: A systematic review and meta-analysis
Source: PLoS One. 2021 Aug 30;16(8):e0256402. doi: 10.1371/journal.pone.0256402 (PMC8405021; doi:10.1371/journal.pone.0256402)
Supplement: S2 File — (PDF) [file pone.0256402.s002.pdf]

Study	Type of study	Was the sample frame appropriate t	Were study participants sampled in an appropriate way?	Was the sample size adequate?	Were the study subjects and the setting described in detail?	Was the data analysis conducted with sufficient coverage of the identified sample?	Were valid methods used for the identification of the condition?	Was the condition measured in a standard, reliable way for all participants?	Was there appropriate statistical analysis?	Was the response rate adequate, and if not, was the low response rate managed appropriately?

		o address the target population?								

Malik et al., 2016	Cross-sectional study	Yes	Yes	Yes	Unclear	Yes	Yes	Yes	Yes	Yes
Pereira-lima et al., 2017	Cross-sectional study	Yes	Yes	Yes	Yes	Yes	Yes	Yes	Yes	Yes
Boni et al., 2018	Cross-sectional study	Yes	Yes	Yes	Yes	Yes	Yes	Yes	Yes	Yes
Kajjimu et al., 2021	Cross-sectional study	Yes	Yes	No	Yes	Yes	Yes	Yes	Yes	Yes
Liu et al., 2018	Cross-sectional study	Yes	Yes	Yes	Yes	No	Yes	Yes	Yes	Yes
Jovanović et al., 2015	Cross-sectional study	Yes	Yes	Yes	Yes	Yes	Yes	Yes	Yes	Yes
Costa et al., 2012	Cross-sectional study	Yes	Yes	Yes	Yes	Unclear	Yes	Yes	Yes	Yes
Asencio-López et al., 2016	Cross-sectional study	Yes	Yes	Yes	Yes	Yes	Yes	Yes	Yes	Yes

Pereira-Lima & Loureiro., 2017	Cross-sectional study	Yes	Yes	Yes	Yes	Yes	Yes	Yes	Yes	Yes
Pokhrel et al., 2020	Cross-sectional study	Yes	Yes	Unclear	No	Yes	Yes	Yes	Yes	Unclear
Almeida et al., 2016	Cross-sectional study	Yes	Yes	Yes	Yes	Yes	Yes	Yes	Yes	Yes
Stein et al., 2016	Cross-sectional study	Yes	Yes	No	Yes	Yes	Yes	Yes	Yes	Yes
Mason & Nel, 2014	Cross-sectional study	Yes	No	Yes	Yes	Yes	Yes	Yes	Yes	No
Mathias et al., 2017	Cross-sectional study	Yes	No	No	Yes	Yes	Yes	Yes	Yes	Yes
Ogoma, 2020	Cross-sectional study	Yes	Yes	No	Yes	Yes	Yes	Yes	Yes	Yes
Bolatov et al. 2021	Cross-sectional study	Yes	Yes	Yes	Yes	Yes	Yes	Yes	Yes	Yes

Daud et al., 2020	Cross-sectional study	Yes	Yes	Yes	Yes	Yes	Yes	Yes	Yes	Yes
Wickramasinghe et al., 2018	Cross-sectional study	Yes	Yes	Yes	Yes	Yes	Yes	Yes	Yes	Yes
Fares et al., 2016	Cross-sectional study	Yes	Yes	Yes	Yes	Yes	Yes	Yes	Yes	Yes
Bolatov et al. 2021	Cross-sectional study	Yes	Yes	Yes	Yes	Yes	Yes	Yes	Yes	Yes
Nikodijević et al., 2012	Cross-sectional study	Yes	Yes	Yes	Yes	Yes	Yes	Yes	Yes	Yes
Barbosa et al., 2018	Cross-sectional study	Yes	Yes	Yes	Yes	Yes	Yes	Yes	Yes	Yes
Fontana et al., 2020	Cross-sectional study	Yes	Yes	Yes	Yes	Yes	Yes	Yes	Yes	Yes
Mafla et al., 2015	Cross-sectional study	Yes	Yes	Yes	Yes	Yes	Yes	Yes	Yes	Yes

Vasconcelos et al., 2020	Cross-sectional study	Yes	Yes	Yes	Yes	Yes	Yes	Yes	Yes	Yes
Calcides et al., 2019	Cross-sectional study	Yes	Yes	Yes	Yes	Yes	Yes	Yes	Yes	Yes
Galdino et al., 2016	Cross-sectional study	Yes	Yes	Yes	Yes	Yes	Yes	Yes	Yes	Yes
Pharasi et al., 2020	Cross-sectional study	Yes	Yes	Yes	Yes	Yes	Yes	Yes	Yes	Yes
Alhaffar et al., 2019	Cross-sectional study	Yes	Yes	Yes	Yes	Yes	Yes	Yes	Yes	Yes
Lopes et al., 2018	Cross-sectional study	Yes	Yes	Yes	Yes	Yes	Yes	Yes	Yes	Yes
Müller et al., 2020	Cross-sectional study	Yes	Yes	Yes	Yes	Yes	Yes	Yes	Yes	Yes
Magri et al., 2019	Cross-sectional study	Yes	Yes	Yes	Yes	Yes	Yes	Yes	Yes	Yes

Tavares et al., 2020	Cross-sectional study	Yes	Yes	Yes	Yes	Yes	Yes	Yes	Yes	Yes
Costa et al., 2012	Cross-sectional study	Yes	Yes	Yes	Yes	Yes	Yes	Yes	Yes	Yes
Wing et al., 2018	Cross-sectional study	Yes	Yes	Yes	Yes	Yes	Yes	Yes	Yes	Yes
Aghajari et al., 2019	Cross-sectional study	Yes	Yes	Yes	Yes	Yes	Yes	Yes	Yes	Yes
Neves et al., 2016	Cross-sectional study	Yes	Yes	Yes	Yes	Yes	Yes	Yes	Yes	Yes
Franco et al., 2011	Cross-sectional study	Yes	Yes	Yes	Yes	Yes	Yes	Yes	Yes	Yes
Tavares et al., 2014	Cross-sectional study	Yes	Yes	Yes	Yes	Yes	Yes	Yes	Yes	Yes
Atlam, 2017	Cross-sectional study	Yes	Yes	Yes	Yes	Yes	Yes	Yes	Yes	Yes

Lee et al., 2020	Cross-sectional study	Ye s	Yes	Yes	Yes	Yes	Yes	Yes	Yes	Yes
Zhang et al., 2021	Cross-sectional study	Ye s	Yes	Yes	Yes	Yes	Yes	Yes	Yes	Yes
Tian et al., 2018	Cross-sectional study	Ye s	Yes	Yes	Yes	Yes	Yes	Yes	Yes	Yes
Pu et al., 2021	Cross-sectional study	Ye s	Yes	Yes	Yes	Yes	Yes	Yes	Yes	Yes
Geng et al., 2020	Cross-sectional study	Ye s	Yes	Yes	Yes	Yes	Yes	Yes	Yes	Yes
Khosravi et al., 2021	Cross-sectional study	Ye s	Yes	Yes	Yes	Yes	Yes	Yes	Yes	Yes
Ji et al., 2020	Cross-sectional study	Ye s	Yes	Yes	Yes	Yes	Yes	Yes	Yes	Yes
Tlilia et al., 2020	Cross-sectional study	Ye s	Yes	Yes	Yes	Yes	Yes	Yes	Yes	Yes

Pharasi et al., 2020	Cross-sectional study	Yes	Yes	Yes	Yes	Yes	Yes	Yes	Yes	Yes
Bera et al., 2013	Cross-sectional study	Yes	Yes	Yes	No	No	No	No	Yes	Yes
Waldman et al., 2009	Cross-sectional study	Yes	Yes	Yes	Yes	Yes	Yes	Yes	Yes	Yes
Martins* et al., 2011	Cohort study	Yes	Yes	Yes	Yes	Yes	Yes	Yes	Yes	Yes
Ogboghodo et al., 2020	Cross-sectional study	Yes	Yes	Yes	Yes	Yes	Yes	Yes	Yes	Yes
Serrano et al., 2016	Cross-sectional study	Yes	Yes	Yes	Yes	Yes	Yes	Yes	Yes	Yes
Parra-Osorio et al., 2015	Cross-sectional study	Yes	Yes	Yes	Yes	Yes	Yes	Yes	Yes	No
Rodrigues et al., 2020	Cross-sectional study	Yes	Yes	Yes	No	No	No	No	Yes	Yes
