# Supplementary figures and images for: Prevalence of burnout among university students in low- and middle-income countries: A systematic review and meta-analysis
Source: PLoS One. 2021 Aug 30;16(8):e0256402. doi: 10.1371/journal.pone.0256402 (PMC8405021; doi:10.1371/journal.pone.0256402)

Funnel plot with pseudo 95% confidence limits

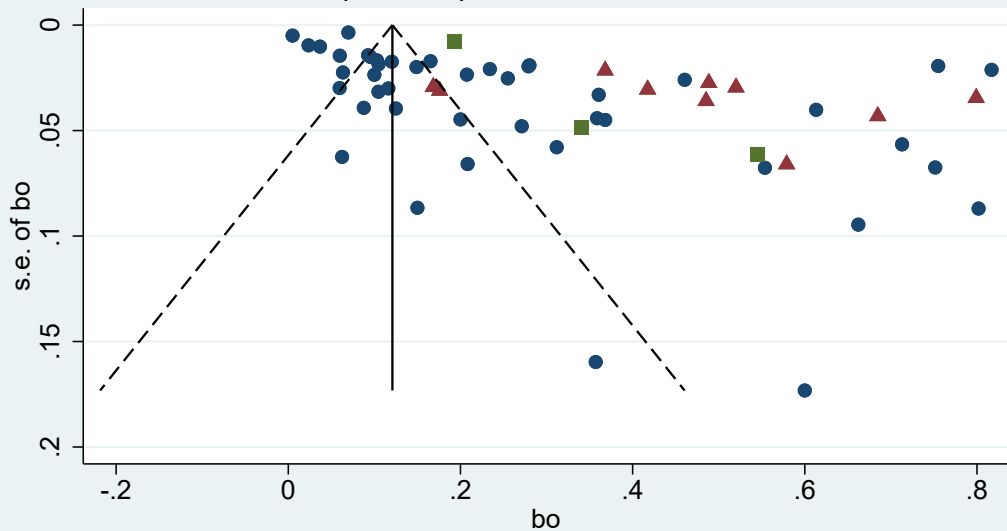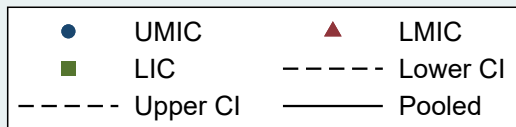

Supplement: S3 File — (PDF) [file pone.0256402.s003.pdf]

Funnel plot with pseudo 95% confidence limits

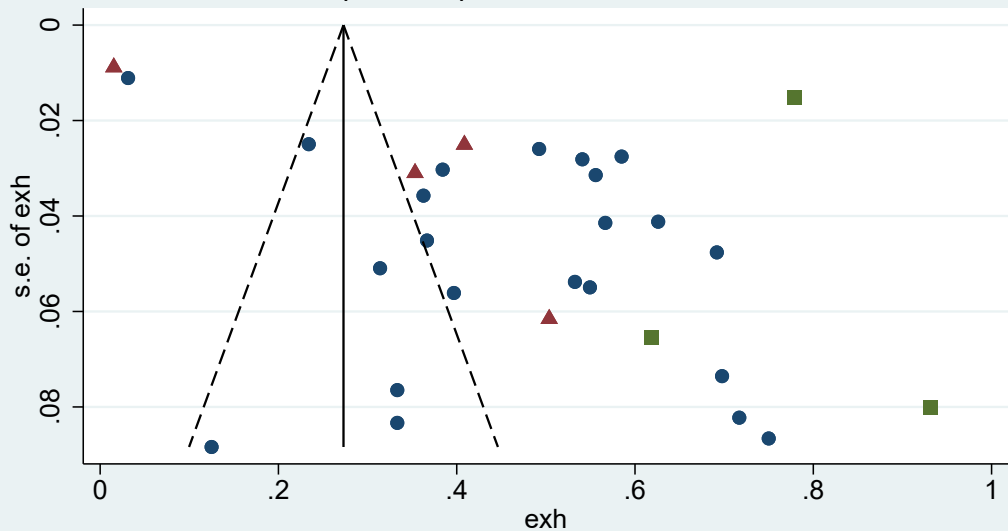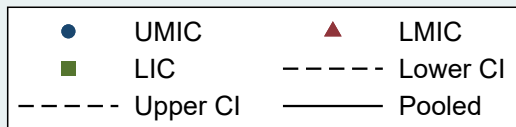

Supplement: S4 File — (PDF) [file pone.0256402.s004.pdf]

Funnel plot with pseudo 95% confidence limits

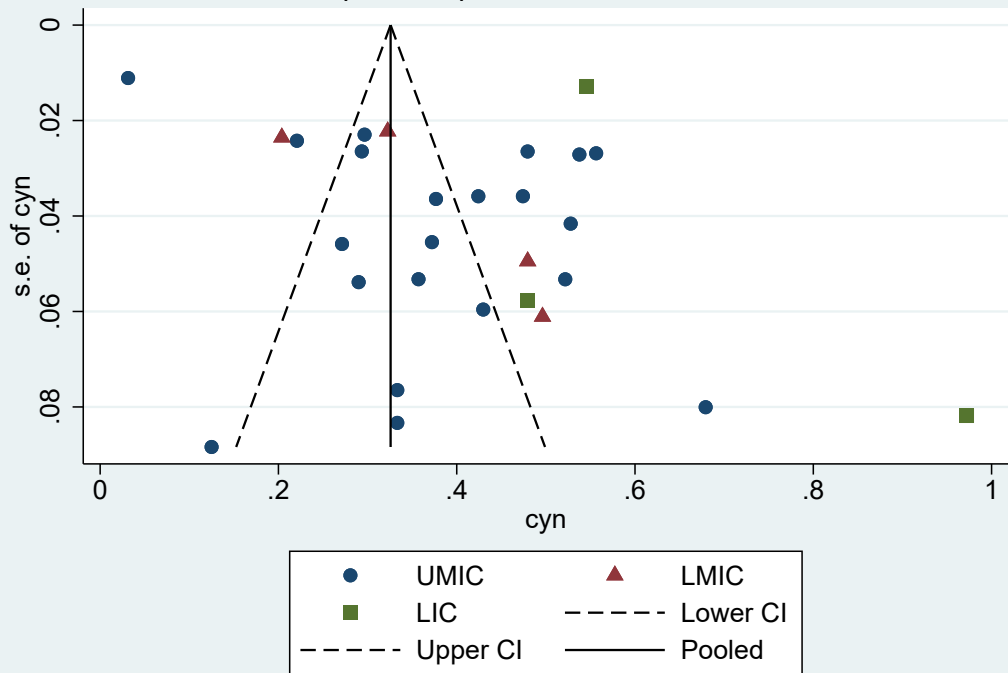

Supplement: S5 File — (PDF) [file pone.0256402.s005.pdf]

Funnel plot with pseudo 95% confidence limits

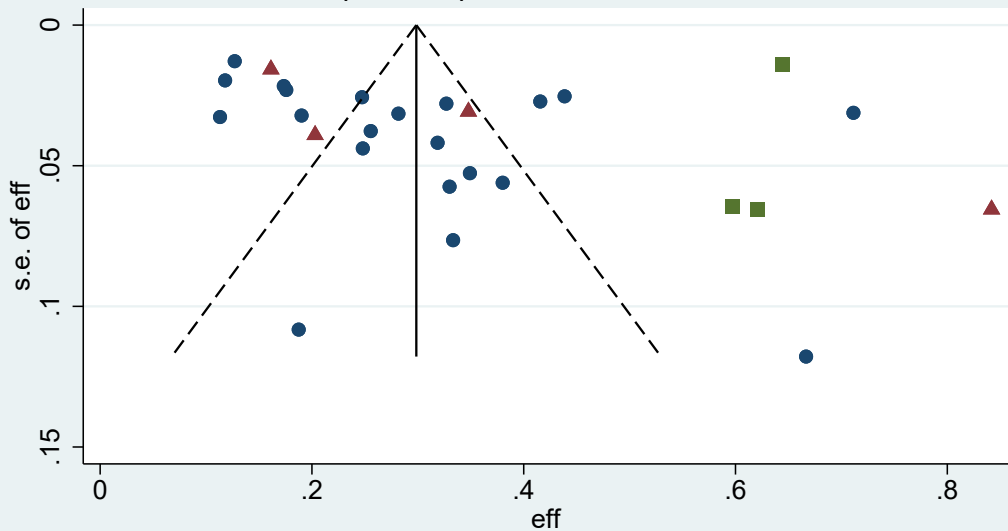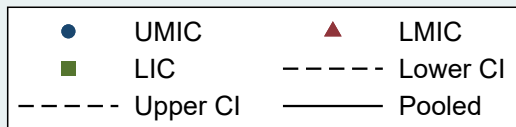

Supplement: S6 File — (PDF) [file pone.0256402.s006.pdf]

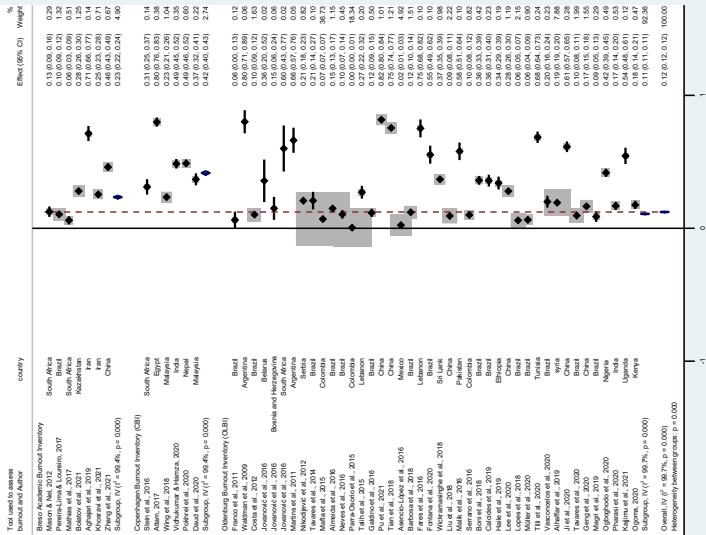

Supplement: S7 File — (PDF) [file pone.0256402.s007.pdf]

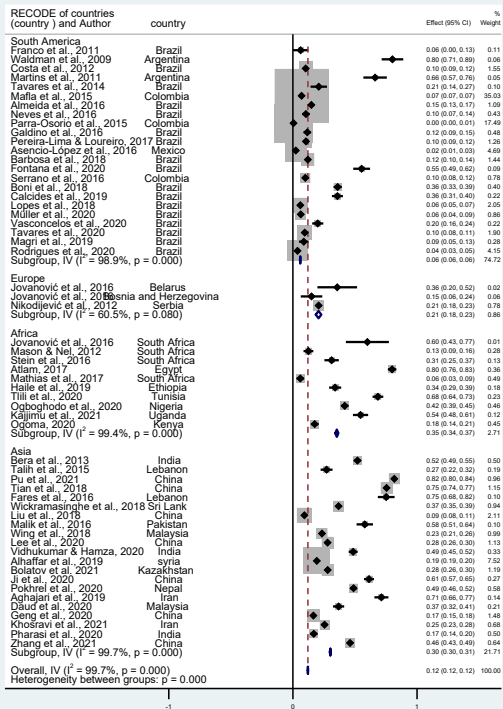

Supplement: S8 File — (PDF) [file pone.0256402.s008.pdf]

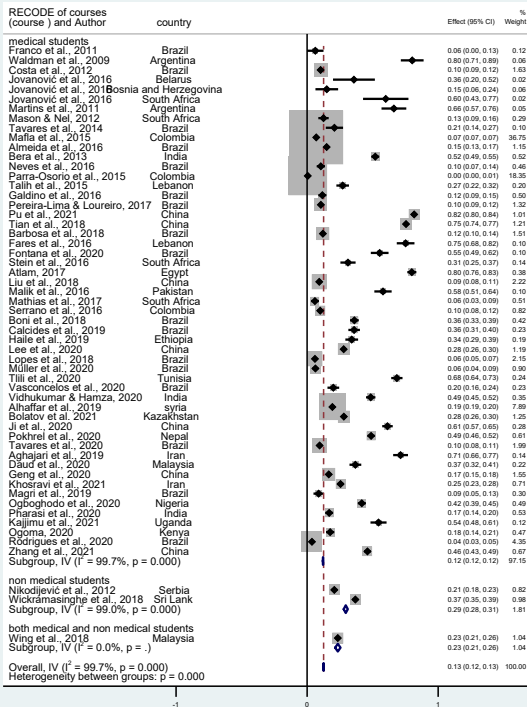

Supplement: S9 File — (PDF) [file pone.0256402.s009.pdf]

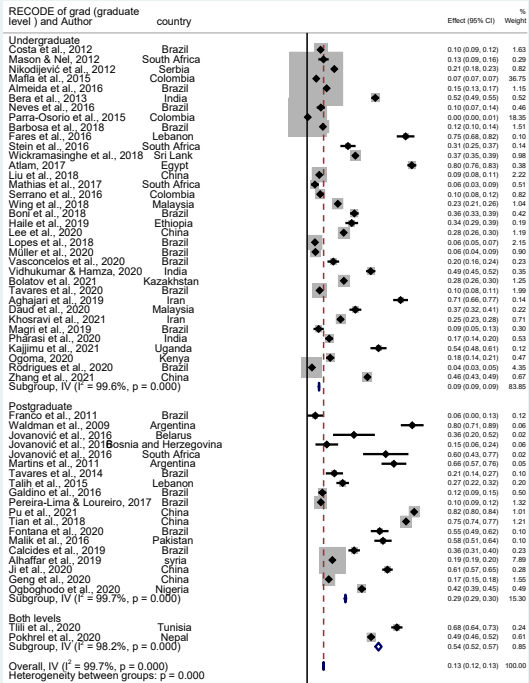

Supplement: S10 File — (PDF) [file pone.0256402.s010.pdf]

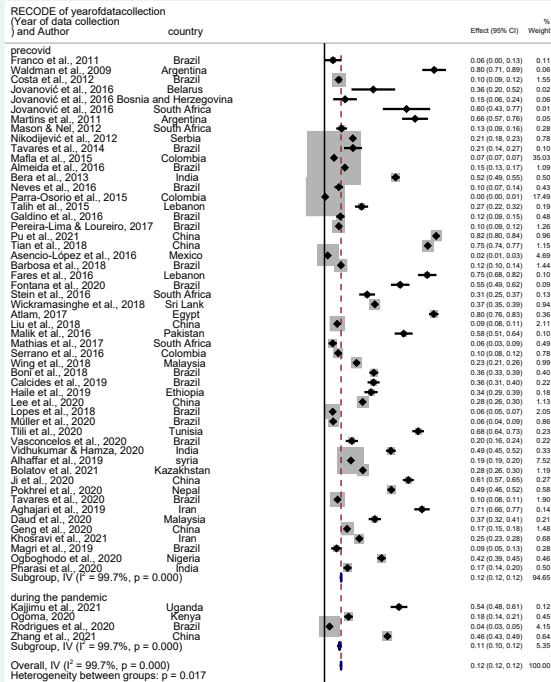

Supplement: S11 File — (PDF) [file pone.0256402.s011.pdf]
