# Supplementary material for: Prevalence of burnout among university students in low- and middle-income countries: A systematic review and meta-analysis
Source: PLoS One. 2021 Aug 30;16(8):e0256402. doi: 10.1371/journal.pone.0256402 (PMC8405021; doi:10.1371/journal.pone.0256402)
Supplement: Attachment — Submitted filename: Response to reviewers .pdf [file pone.0256402.s014.pdf]

Jonathan Kajjimu
Mbarara University of Science and Technology
P.O.Box 1410,
Mbarara – Uganda.
June 24, 2021

Academic Editor,

PLOS ONE

Dear Dr. Sabeena Jalal

RE: RESPONSES TO REVIEWERS' COMMENTS

Editor's comments

1. Please correct the numbers in the PRISMA Flow Diagram. The numbers are not adding up. 2214 - 1886 = 328, not 258.

Our response:

Thanks for this keen observation to identify this issue. We had accidentally left out of the PRISMA flow diagram other papers (n=70) which we hadn't categorized.

2. 2) Please add a definition of LMIC, and mention which criterion for LMIC distribution did you use. If it was the World Bank, and please add it in the description

Our response:

In the Methods section under the search strategy, we have added a LMIC definition of "Low and Middle Income Countries" according to the World Bank Country and Lending Groups, 2021. (reference 20)

Journal requirements:

1. Please ensure that your manuscript meets PLOS ONE's style requirements, including those for file naming. The PLOS ONE style templates can be found at https://journals.plos.org/plosone/s/file?id=wjVg/PLOSOne_formatting_sample_main_body.pdf and https://journals.plos.org/plosone/s/file?id=ba62/PLOSOne_formatting_sample_title_authors_affiliations.pdf

Our response:

We have used these templates to make edits to our manuscript to enable it meet PLOS ONE's style requirements.

2. Please amend the manuscript submission data (via Edit Submission) to include authorLetizia Maria Atim.

Our response:

We have added Letizia Maria Atim as a coauthor.

Reviewer 1:

4. Is the manuscript presented in an intelligible fashion and written in standard English?

PLOS ONE does not copyedit accepted manuscripts, so the language in submitted articles must be clear, correct, and unambiguous. Any typographical or grammatical errors should be corrected at revision, so please note any specific errors here.

Reviewer #1: No

Our response:

Our revised manuscript has been copy edited by a coauthor of the paper who is a native English speaker, called Dr. Andrew Tagg.

Comments in attached file.

Our response:

We have responded to all comments in the attached file.

Reviewer 2:

1. Please add stratified results as suggested below:
   a. Region
   b. Field of study and
   c. Level of study (grad vs undergrad) to address the limitation the authors mentioned. Individual study results are not necessary, an overview table with aggregate results including basic information such as number of studies and prevalence would suffice. Please also add relevant methods and discussion.

Our response:

We have included additional stratifications of our study data basing on the region, field of study, and level of study. Forest plots of these stratifications have also been supplied as supplementary files 9,10, and 11 respectively.

2. Consider stratifying pre and during Covid19, if sufficient studies are available to separately look at this time period and add a brief discussion.

Our response:

We have added a stratification of our data based on findings pre and during COVID-19. A supplementary file12 of a forest plot of this stratification has also been provided.

Many thanks in advance for your time and kind consideration.

Yours sincerely,
Jonathan Kajjimu
