# Supplementary material for: Prevalence of burnout among university students in low- and middle-income countries: A systematic review and meta-analysis
Source: PLoS One. 2021 Aug 30;16(8):e0256402. doi: 10.1371/journal.pone.0256402 (PMC8405021; doi:10.1371/journal.pone.0256402)
Supplement: Attachment — Submitted filename: reviewed_Burnout systematic review final.docx [file pone.0256402.s013.docx]

**Prevalence of burnout among university students in low- and middle-income countries: A systematic review and meta-analysis**

Mark Mohan Kaggwa ^1^, Jonathan Kajjimu ^2*^, Jonathan Sserunkuma ^2^, Sarah Maria Najjuka ^3^, Letizia Maria Atim ^1^, Ronald Olum ^3^, Andrew Tagg ^4, 5,^ and Felix Bongomin ^6^

**Affiliations**

^1^ Department of Psychiatry, Faculty of Medicine, Mbarara University of Science and Technology, Mbarara, Uganda

^2^ Faculty of Medicine, Mbarara University of Science and Technology, Mbarara, Uganda

^3^ College of Health Science, Makerere University, Kampala, Uganda

^4^ Emergency Department, Western Hospital-Footscray, Footscray, Victoria, Australia

^5^ School of Medicine, University of Melbourne, Melbourne, Victoria, Australia

^6^ Department of Medical Microbiology and Immunology, Faculty of Medicine, Gulu University, Gulu, Uganda

***Corresponding Author**

Jonathan Kajjimu

P. O. Box 1410, Mbarara, Uganda

Email: jonathkebenz37@gmail.com

**Abstract**

**Background**

Burnout is common among university students worldwide and may adversely affect their academic performance. Little is known about the true burden of this preventable malady among university students in low-and-middle-income countries (LMICs).

**Objectives**

This study aimed to systematically estimate the prevalence of burnout among university students in LMICs.

**Methods**

PubMed, Google Scholar, CINAHL, Web of Science, African Journals Online, and Embase were searched from the inception of each database until February 2021. Original studies were included. No study design or language restrictions were applied. A random-effects meta-analysis was performed using STATA version 16.0. Heterogeneity and publication bias were assessed using Q-statistics and funnel plots, respectively.

**Results**

Fifty-five unique articles, including a total of 27,940 (Female: 16,215, 58.0%) university students from 24 LMICs were included. The Maslach Burnout Inventory (MBI) was used in 43 studies (78.2%). The pooled prevalence of burnout was 12.1% (95% Confidence Interval (CI) 11.9 – 12.3; I^2^ = 99.7%, Q = 21,464.1, P-value = < 0.0001). The pooled prevalence of emotional exhaustion (feelings of energy depletion), cynicism (negativism), and reduced professional efficacy were, 27.8% (95% CI 27.4 – 28.3; I^2^ = 98.17%. P-value = <0.0001), 32.6 (95% CI: 32.0 – 33.1; I^2^: 99.5%; P-value= < 0.0001), and 29.9% (95% CI: 28.8 – 30.9; I^2^: 98.1%; P-value = < 0.0001), respectively.

**Conclusion**

Nearly one-third of university students in LMICs experience burnout. More studies are needed to understand the causes of burnout in this key population. There is a need to validate freely available tools for use in these countries.

**Keywords:** Burnout, features of burnout, components of burnout, LMICs, burnout assessment tools, MBI, burnout associated factors**,** university students, medical students, medical residents

1. **Introduction**

Burnout is a psychological syndrome that may arise as a response to chronic interpersonal stressors at work ^1^. Burnout is characterized by feelings of energy depletion or emotional exhaustion (EE) - feelings of exhaustion caused by educational demands, increased mental distance from one’s studies, and feelings of cynicism related to one’s studies/job, coupled with reduced personal/professional efficacy (PE) - the feeling of incompetence as a student ^2^. Burnout can occur to anyone involved in a psychologically engaging activity like higher education ^3^.

University education is an intrinsically demanding stage in many students' lives ^4,5^. There are several demands on a student’s time including course work, relationships, examinations, part-time work, internship, pressure from parents and guardians, and practical/ward work for medical students ^4,5^. This puts many students at risk of burnout ^4^. The literature has grouped burnout risk factors into 3 groups: **individual factors**, including sociodemographic variables; **education characteristics**, such as workload, time pressure, the course offered, part-time work, and emotional demands such as relationships; **university characteristics**, which include hierarchies, operating rules, resources, values, management model, culture, psychological support, and curricular factors ^6-8^. Burnout is associated with poor academic performance, sleep disturbance, risk of severe mental illness or substance use disorder, an increased likelihood of cardiovascular disease, and neglect of physical and mental health ^5,9^.

There have been disparities between recent studies () regarding the prevalence of burnout. In high-income countries (HICs), such as Saudi Arabia, the prevalence was noted 30.5% in a population of students doing medical-related courses. In a similar cohort in Uganda, the prevalence was 54.5% ^10,11^.

Discrepancies in prevalence rates were attributed to different exposures to some socio-economic, political, health, and conflict-related stress factors ^12^. Systematic reviews on burnout have been performed among students doing medical-related programs, predominantly in high-income countries (HICs) ^13-16^. These overshadow the findings of students’ wellness and burnout in low- and middle-income countries (LMICs). The few reviews done in LMICs are from middle-income countries (MICs) and have all been among students pursuing medical-related courses ^17^.

In this study, the research aimed to provide an insight into the burden of burnout syndrome among university students in LMICs by conducting a systematic review and meta-analysis to evaluate the prevalence of burnout and its different sub-components.

2. **Methods**

**Study design**

The researchers used the Meta-analysis of Observational Studies in Epidemiology (MOOSE) guidelines for systematic review and meta-analysis of observational studies ^18^, in addition to the Preferred Reporting Items for Systematic Reviews and Meta-Analyses (PRISMA) guidelines ^19^. The study protocol was registered with PROSPERO (CRD42021232487).

**Search strategy**

With the help of a qualified medical librarian, relevant databases (PubMed, Google Scholar, CINAHL, Web of Science, African Journals Online, and Embase) were used for literature search, from the inception of each database until February 15^th,^ 2021. The search strings used were; burnout, burned out, emotional exhaustion, compassion fatigue, excess depersonalization, personal accomplishment, personal burnout, studies-related burnout, colleague related burnout, teacher-related burnout. Also, prevalence, burden, incidence, University Students, college students, Medical Students, and the list of all countries in LMICs^20^. (**Supplementary file 1**). Additional articles were obtained from manual search of the references of the selected articles. The corresponding authors whose articles were not freely available were contacted telephonically and via emails.

The articles included were published peer-reviewed articles of all languages, around the prevalence of burnout among university students in LMICs. Cross-sectional, cohort, and case-control studies were included. Review articles, single case reports, and small case series were excluded.

**Study selection process**

All identified eligible articles were imported into Endnote 9 to ascertain duplicates. After removal of duplicates, article selection by title and then abstract was performed by two independent reviewers (LMA and JK). Any discrepancy in the included articles was settled by MMK. Articles were included for full article review by MMK and FB. The remaining articles were included for qualitative and quantitative synthesis checks. These articles were divided into pairs among (LMA and JS) and (JK and SMN). Any disagreement among the individuals was settled by the lead investigator (MMK), (**Figure 1)**.

**Data management and extraction**

A pre-piloted google form was used for data extraction. The following information were captured: The first authors, title, year of data collection, country, sample size, individuals with burnout and features of burnout, age, sex, year of study, the tool used, and study population.

**Quality assessment**

The Joanna Briggs Institute (JBI) checklist for prevalence studies ^21^ was used to evaluate the risk of bias of the included articles. JBI uses a 4-point Likert scale with answers being “no”, “yes”, “unclear”, or “not applicable”, for the following questions (1) appropriateness of the sample frame; (2) recruitment procedure; (3) adequacy of the sample size; (4) description of subjects and setting; (5) description of the identified sample; (6) validity of the methods used to screen for burnout; (7) reliability of the methods used to screen for burnout; (8) adequacy of statistical analyses; and (9) response rate. Articles were assigned one point per yes. Articles with a score less than 5 were excluded. All the selected articles were included for further synthesis. The results are presented in **Supplementary file 2**.

**Data analysis**

Based on the number of students who had burnout or its different features, a random effect meta-analysis was performed using STATA version 16.0 (StataCorp, College Station, TX, USA). The Q statistics and I^2^ accounted for the heterogeneity among the studies ^22^. A random-effects model was used to determine the overall pooled estimates of effect sizes (prevalence), when heterogeneity was > 50, the researchers used fixed-effect models when the heterogeneity was lower. The results were presented on forest plots, the Funnel plots were generated to visually assess for publication bias. The study tools were summarized and a comparison between the different tools made concerning the relation to pooled burnout prevalence. P-values were two-tailed, and were considered statistically significant if the P-value was ≤ 0.05.

3. **Results**

A total of 2245 studies were retrieved. Of these, 55 articles including a total of 28,206 **(**Male - 11,121 and Female - 16,398) university students and published between the year 2006 to 2020 met our inclusion criteria. Most of the studies were conducted in Brazil (n = 18), and China (n = 7). Many of the studies were from upper-middle-income countries (n = 45), with 10 from lower-middle-income countries and 3 from lower-income countries. A total of 3 studies were conducted among students undertaking non-medical related programs, and 21 studies were conducted among postgraduate students. Participants took place from the breadth of their training program. For details about the participants and study characteristics see **Table 1.**

**Table 1: Characteristics of the studies**

| **Author** | **Country** | **Year of data collection** | **Sample Size** | **Gender Male**  **Female** | **Age** | **Years of study** | **Study population** | **burnout**  **n (%)** | **Emotional exhaustion**  **n (%)** | **Cynicism n (%)** | **Reduced professional efficiency n (%)** | **The tool used to assess burnout** |
| --- | --- | --- | --- | --- | --- | --- | --- | --- | --- | --- | --- | --- |
| Franco et al., 2011 ^23^ | Brazil | 2004 | 16 | 1    15 | 25.8 |  | P, M | 1 | 2 | 2 | 3 | MBI |
| Waldman et al., 2009 ^24^ | Argentina | 2007 | 106 | 70    36 | 29.1 |  | P, M | 85 | 76 | 72 | 12 | MBI |
| Costa et al., 2012 ^3^ | Brazil | 2009 | 369 | 186    183 |  |  | U, M | 38 | 231 | 175 | 64 | MBI |
| Jovanović et al., 2016 ^25^ | Belarus | 2009 | 14 | 2    12 | 25.1(1.1) |  | P, M | 5 |  |  |  | MBI |
| Jovanović et al., 2016 ^25^ | Bosnia and Herzegovina | 2009 | 20 | 6    14 | 33.6(3.4) |  | P, M | 3 |  |  |  | MBI |
| Jovanović et al., 2016 ^25^ | South Africa | 2010 | 20 | 9  11 | 31.8(5.9) |  | P, M | 12 |  |  |  | MBI |
| Martins et al., 2011 ^26^ | Argentina | 2011 | 74 | 14    60 |  |  | P, M | 49 |  |  |  | MBI |
| Nikodijević et al., 2012 ^27^ | Serbia | 2011 | 376 | 159    217 | - | - | U, N | 78 | 88 | 83 | 93 | MBI |
| Tavares et al., 2014 ^28^ | Brazil | 2011 | 48 | 4    44 | 26(2.9) | Year 2 (48) | P, M | 10 | 16 | 16 | 32 | MBI |
| Mason & Nel, 2012 ^29^ | South Africa | 2011 | 80 | 7    73 | 22.4 | year 1 (33), year 2 (24), year 3 (23) | U, M | 10 |  |  |  | ProQOL R-IV |
| Mafla et al., 2015 ^30^ | Colombia | 2012 | 5647 | 1719    3928 | Range 18 – 24; Under 18 (665), 18 – 21(2371), 22 – 24 (1793), >24 (818) | Year 1 (1348), Year 2 (1294), Year 3 (1178), Year 4 (1144), & Year 5 (683) | U, M | 394 |  |  |  | MBI |
| Neves et al., 2016 ^31^ | Brazil | 2013 | 105 |  | 21.25(2.53) |  | U, M | 11 |  |  |  | MBI |
| Talih et al., 2016 ^32^ | Lebanon | 2013 | 118 | 62    56 | 18 -25(26), 26 - 35 (92) | Year 1 (33), Year 2 (31), Year 3 (26), Year 4 (28) | P, M | 32 |  |  |  | MBI |
| Bera et al., 2013 ^33^ | India | 2013 | 596 | 529    67 |  |  | U, M | 310 |  |  |  | Not clear |
| Almeida et al., 2016 ^34^ | Brazil | 2013 | 376 | 151    216 | <21(119), 21 - 25 (209), 26> (39) | Year 1 (101), Year 2 (76), Year 3(100), Year 4(89) | U, M | 56 |  |  |  | MBI |
| Parra-Osorio et al., 2015 ^35^ | Colombia | 2013 | 201 | 90    111 | 20.9 (2.9) |  | U, M | 1 |  |  |  | MBI |
| Pereira-Lima et al., 2017 ^36^ | Brazil | 2014 | 305 | 159    146 | 28(2.530) | Year 1& 2 (156), Year 3 - 5 (149) | P, M | 32 | 211 | 161 | 36 | OLBI |
| Galdino et al., 2016 ^37^ | Brazil | 2014 | 129 | 13    116 | 32.3 | Masters (79), Ph.D. (50), | P, M | 15 | 90 | 35 | 32 | MBI |
| Tian et al., 2019 ^38^ | China | 2014 | 2008 | 593    1218 |  | Masters’ Year 1 (391), Master’s Year 2 (554), Master’s Year 3 (609), Ph.D. Year 1 (79), Ph.D. Year 2 (73), Ph.D.hD Year 3 (68) | P, M | 1516 |  |  |  | MBI |
| Pu et al., 2021 ^39^ | China | 2014 | 1814 | 596    1218 |  | Masters’ Year 1 (391), Master's Year 2 (554), Master’s Year 3 (609), Ph.D. Year 1 (79), Ph.D. Year 2 (73), & Ph.D. Year 3 (68) | P, M | 1482 |  |  |  | MBI |
| Stein et al., 2016 ^40^ | South Africa | 2015 | 93 |  |  | Year 4(93) | U, M | 29 |  |  |  | CBI |
| Barbosa et al., 2018 ^41^ | Brazil | 2015 | 399 | 177    222 | 21(3.6) | - | U, M | 48 |  |  |  | MBI |
| Fares et al., 2016 ^12^ | Lebanon | 2015 | 165 | 88    77 | 18 – 24 (161)  >25 (4) | Year 1 (80), year 2 (85) | U, M | 124 |  |  |  | MBI |
| Wickramasinghe et al., 2018 ^42^ | Sri Lank | 2015 | 796 | 356    440 | 18.4 (0.32) | - | U, N | 293 |  |  |  | MBI |
| Fontana et al., 2020 ^43^ | Brazil | 2015 | 121 | 68    53 | 25 | Year 1 (33) & year 2 (35) | P, M | 67 | 38 | 52 | 46 | MBI |
| Liu et al., 2018 ^44^ | China | 2016 | 453 | 199    254 | 20.21(1.46) | Year1(129), Year2(27)  Year3(280), Year4(16), Year5(1) | U, M | 42 |  |  |  | MBI |
| Malik et al., 2016 ^45^ | Pakistan | 2016 | 133 | 98  35 |  | Year1(46), Year2(45), Year3(22), Year4(19) | P, M | 77 | 67 | 66 | 27 | MBI |
| Wing et al., 2018 ^5^ | Malaysia | 2016 | 538 | 312    226 | 22.3 (1.3) |  | U, M, N | 126 |  |  |  | CBI |
| Mathias et al., 2017 ^46^ | South Africa | 2016 | 67 | 13    54 | 20-24(57), 25-29(5), 30-34(4), >35(1) | Year 3 (26),  Year 4 (41) | U, M | 4 |  |  |  | ProQOL R-IV |
| Atlam, 2018 ^47^ | Egypt | 2016 | 672 | 232    440 | <22 (411), >21 (261) |  | U, M | 537 |  |  |  | CBI |
| Serrano et al., 2016 ^48^ | Colombia | 2016 | 180 | 94    86 | 20(19 – 22) |  | U, M | 18 | 66 | 67 | 46 | MBI |
| Lee et al., 2020 ^49^ | China | 2017 | 731 | 323    408 | 20.54 (2.07) | Year 1 (118),  Year 2 (289),  Year 3 (90),  Year 4 (148),  Year 5 (167), & Year 6 (37) | U, M | 204 | 360 | 393 | 520 | MBI |
| Calcides et al., 2019 ^50^ | Brazil | 2017 | 184 | 83    101 | 25.9(3.9) |  | P, M | 66 | 98 | 96 | 35 | MBI |
| Haile et al., 2019 ^51^ | Ethiopia | 2017 | 144 | 123    98 | 30(3) | Year 1 (17),  Year 2 (28),  Year 3 (28),  Year 4 (39),  Year 5 (32) | U, M | 49 | 89 | 69 | 86 | MBI |
| Lopes et al., 2020 ^52^ | Brazil | 2017 | 284 | 28    256 |  |  | U, M | 17 | 103 | 107 | 80 | MBI |
| Tlili et al., 2021 ^53^ | Tunisia | 2017 | 368 | 49    319 |  | Masters (40), others (328) | P, U, M | 252 | 130 | 75 | 128 | MBI |
| Vidhukumar & Hamza, 2020 ^54^ | India | 2017 | 375 | 142    233 |  | Year 2 (60),  Year 3 (73),  Year 4 (34),  Year 5 (44), & Interns (60) | U, M | 182 |  |  |  | CBI |
| Vasconcelos et al., 2020 ^55^ | Brazil | 2017 | 100 | 9    91 | 18 – 27 (87), 27 -57 (13) | Year 1 (36),  Year 2 (16),  Year 3 (24), & Year 4 (24) | U, M | 20 | 75 | 29 | 33 | MBI |
| Boni et al., 2018 ^56^ | Brazil | 2017 | 330 | 96    183 | 21.4(2.7) | Year1(118), Year2(59), Year3 (51), Year4 (49) | U, M | 119 | 187 | 140 | 58 | MBI |
| Müller et al., 2020 ^57^ | Brazil | 2017 | 126 | 53    73 | 18 – 20 (32), 21 – 25 (81), 26 – 30 (11), & > 31 (2) | Year 1 (32),  Year 2 (28),  Year 3 (27), & Year (39) | U, M | 8 | 50 | 45 | 44 | MBI |
| Alhaffar et al., 2019 ^58^ | Syria | 2018 | 3350 | 1477    1873 | 21–25 (1139), 26 – 30 (1994), 31 – 35 (217) | Year 1 (1311), Year 2 (887),  Year 3 (510),  Year 4 (358), & Year 5 (284) | P, M | 646 | 2609 | 1829 | 2160 | MBI |
| Tavares et al., 2020 ^59^ | Brazil | 2018 | 419 | 148    271 | 22.1(4.3) |  | U, M | 40 | 161 | 123 | 137 | MBI |
| Ji et al., 2020 ^60^ | China | 2018 | 380 | 79    301 | 26 – 29 (214), >29 (166) | Year 1 & 2 (160), Year 3 (73),  Year 4 & 5 (147) | P, M | 233 |  |  |  | MBI |
| Bolatov et al. 2021 ^61^ | Kazakhstan | 2018 | 771 | 193    578 | 20.7 | Year 1 (218), Year 2 (137), Year 3 (125), Year 4 (62), Year 5 (60), & Year 6 (169) | U, M | 216 | 451 | 429 | 98 | CBI |
| Daud et al., 2020 ^62^ | Malaysia | 2019 | 182 | 53    129 |  | Year 1 (37), Year 2 (40), Year 3 (33), Year 4 (35), & Year 5 (37) | U, M | 67 | 100 |  | 58 | CBI |
| Magri et al., 2019 ^63^ | Brazil | 2019 | 57 | 18    39 | 23(2,5) |  | U, M | 5 | 19 | 19 | 19 | MBI |
| Pharasi et al., 2020 ^8^ | India | 2019 | 196 | 133    63 | 20.35 (1.50) | Year 1 (48), Year 2 (50), Year 3 (50), Year 4 (48) | U, M | 33 | 3 | 94 | 165 | MBI |
| Pokhrel et al., 2020 ^64^ | Nepal | 2019 | 651 | 496    156 | 25(4) | Year 1(66), Year2 (68), Year3 (71), Year4 (63), Year5 (63) | U, P, M | 318 | 266 | 210 | 105 | CBI |
| Aghajari et al., 2019 ^65^ | Iran | 2019 | 223 |  |  |  | U, M | 159 |  |  |  | BABI |
| Geng et al., 2020 ^66^ | China | 2019 | 563 | 221    342 | <27 (215), >26 (348) |  | P, M | 93 | 313 | 167 | 234 | MBI |
| Khosravi et al., 2021 ^67^ | Iran | 2019 | 400 | 156    244 | <24 (222), >23 (178) | Year 1 (68), Year 2 (58), Year 3 (80), Year 4 (77), & Others (117) | U, M | 102 |  |  |  | BABI |
| Ogboghodo et al., 2020 ^68^ | Nigeria | 2019 | 448 | 279    169 | 33.9(4.0) | Year 1 (111), Year 2 (138), Year 3 (94), Year 4 (105) | P, M | 187 |  |  |  | MBI |
| Kajjimu et al., 2021 ^11^ | Uganda | 2020 | 145 | 102    43 | 18- 23 (91), 24 - 40 (54) | Year 1(22), Year 2(28), Year 3 (38), Year 4 (26), Year 5 (31) | U, M | 79 | 135 | 141 | 90 | MBI |
| Zhang et al., 2021 ^69^ | China | 2020 | 684 | 290    234 | 20(17 – 24) | Year 2 (310), Year 3 (234), Year 4 (& above139) | U, M | 315 | 370 | 328 | 300 | LBS |
| Ogoma, 2020 ^70^ | Kenya | 2020 | 182 | 98    78 | 26.4(8.28) | Year 2 (95), Year 4 (50), Year 6 (37) | U, M | 32 |  |  |  | MBI |
| Rodrigues et al., 2020 ^86^ | Brazil | 2020 | 350 | 160    169 |  | Year 1 (66), Year 2 (62), Year 3 (57), Year 4 (63), Year 5 (50), Year 6 (55) | U, M | 13 |  |  |  | MBI |
| Total |  |  | **27940** | **10935**  **16215** |  |  |  | **8966** | **6412** | **5031** | **4741** |  |

P = Postgraduate, U = Undergraduate, M = medical-related programs, N = Non-medical related programs, MBI = The Maslach Burnout Inventory, BABI = Breso Academic Burnout Inventory, CBI = Copenhagen Burnout Inventory, ProQOL R-IV = The fourth revision of the Professional Quality of Life Scale, OLBI = Oldenburg Burnout Inventory, and LBS = Learning Burnout Scale

**Prevalence of burnout**

The prevalence of burnout was reported in 55 studies. With 8,966 participants in LMICs had burnout syndrome. The pooled prevalence, from the 24 countries, was 12.1% (95% CI 11.9 – 12.3; I^2^ = 99.7%, Q = 21,464.1, P value = <0.0001). There was marked heterogeneity between studies. The pooled prevalence in Upper Middle Income countries (UMIC), Lower middle income countries (LMIC), and Lower income countries was 9.8% (95% CI 9.6 – 10.0; I^2^ = 99.7%, Q = 15444.6, P-value <0.0001), 42.9% (95% CI 41.9 – 43.9; I^2^ = 99.3%, Q = 1251.5, P-value <0.0001), and 20.1% (95% CI 19.4 – 20.9; I^2^ = 98.7%, Q = 156.8, P-value <0.0001), respectively. Figure 2. The funnel plot by countries’ income status is presented in supplementary file 3. Publication bias is seen especially among studies done in LMIC and LIC

**The prevalence of emotional exhaustion/feelings of energy depletion in LMICs**

A total of 6,412 students had exhaustion, over 29 studies. The pooled prevalence was 27.8% (95% CI 27.4 – 28.3). There was significantly (P-value <0.0001) high level of heterogeneity I^2^ = 98.17%. and Q = 12,756.3. The pooled prevalence in UMIC, LMIC, and LIC was 30.1% (95% CI 29.4 – 30.7; I^2^ = 99.8%, Q = 4,642.7, P-value <0.0001), 8.6% (95% CI 7.8 – 9.4; I^2^ = 99.8%, Q = 1,345.6, P-value <0.0001), and 77.6% (95% CI 76.1 – 79.0; I^2^ = 94.6%, Q = 36.8, P-value <0.0001), respectively. Figure 3 shows the forest plot of the 30 studies and its funnel plot in supplementary file 4.

**The prevalence of cynicism/negativism in LMICs**

From 28 studies, conducted in 14 countries, 5,031 participants displayed high cynicism. The pooled prevalence was 32.6 (95% CI: 32.0 – 33.1; I^2^: 99.5%; Q = 5381.3, P-value<0.0001) The forest plot in Figure 4 shows the distribution of the study prevalence’s. Supplementary 5 shows a figure of the funnel plot of the studies.

**The prevalence of reduced professional efficacy in LMICs**

A total of 4,741 students had professional efficacy from 29 studies. The pooled prevalence was 29.9% (95% CI: 28.8 – 30.9; I^2^ = 98.1%; Q = 1436.3, p<0.0001). There was a high level of heterogeneity. Figure 5 shows the forest plot and accompanying funnel plot in supplementary figure 6.

**The tools used to assess for burnout in LMICs**

The most commonly used tool was the Maslach Burnout Inventory (MBI) (n = 42), followed by the Copenhagen Burnout Inventory (CBI) (n = 6). The Breso Academic Burnout Inventory and the fourth revision of the Professional Quality of Life (ProQOL R-IV) Scale were each used in 2 studies. The Oldenburg Burnout Inventory (OLBI), Learning Burnout Questionnaire, and the Russian version of the Copenhagen Burnout Inventory - Student Survey (R-CBI-S) were each used in one study. The methods are shown in Table 1

Disaggregated by the tool used, the pooled prevalence of burnout was 10.8 % (95% CI: 10.6 – 11.0; I^2^: 99.7%; Q = 16,051.5; p = <0.0001) using MBI, 41.6% (95% CI 40.3 – 42.9; I^2^ :99.4%; Q = 823.7, p= <0.0001) using CBI; and 23.5 (95% CI 22.5 – 24.4: I^2^= 99.4; Q = 939.8, p= <0.0001) using other tools. Forest plot in supplementary file 7

**Discussion**

Conducted with a perspective of student wellness in LMICs, the objective of this systematic review and meta-analysis was to collate data surrounding the burden of burnout in university students. The hope is that this could foster the implementation of evidence-based programs to combat burnout among students. To our knowledge, no other study has reviewed LMICs, and hence this study provides a great insight into levels of burnout, the tools used, and factors that could impact a student’s productivity, efficiency, quality, and academic performance. The results indicate low levels of burnout (12.0%), emotional exhaustion (27.8%), cynicism (32.6%), and reduced professional efficacy (29.9%). These findings suggest lower rates than those found in high income country students as well as medical residents (26.8% - 43.5%) or emergency medicine residents (55.6% – 77.9%), scoring for general burnout and 31.8% - 46.0% for emotional exhaustion ^15,71,72^. This high level of discrepancy may be due to medical students experiencing both work-related stress and academic stress, but our synthesis had non-medical students who had lower levels of burnout. Our findings showed a marked publication bias. This could be a reason for the low levels of burnout reported in the region, as a result of few publications on the burnout aspects ^73^. The findings were mainly from middle-income countries (MICs) (Brazil and China) in which burnout has been studied. Public awareness about its consequences is known ^72,74-76^. The low-income countries (LICs), which mainly reported a higher prevalence of burnout and its components ^11,25^, need to preach more about burnout. Increasing the number of studies with country and cultural-based interventions to reduce burnout ^77^ This unevenly distributed knowledge surrounding burnout may be related to the expensive study tools used in screening, precluding use in LICs.

The MBI is most used due to its good psychometric properties. It has been validated across many different cultures and countries ^78^. Based on our synthesis, the prevalence of burnout among students, using the MBI tools, is statistically lower than that reported using other tools among other groups such as health workers ^79^. The reliability of its findings appears comparable across different countries ^78^, but it currently remains the only tool that requires payment for use - a major hindrance in LMICs ^80^. Countries should validate the freely available tools to have comparable results to those studies using the MBI tools. The shift to other tools may increase knowledge and awareness about the burnout syndrome among the LMICs so that appropriate interventions can be implemented.

This review shows that the level of burnout in LICs is higher compared to middle-income countries' economic levels among the LMICs. Probably previously proved low-cost interventions among health workers should be used among students to enhance coping skills and mitigate education-related distress and burnout ^81-83^. They include mindfulness practices, yoga exercises, and group discussions where individuals connect and share their experiences ^81-84^. These are low-cost interventions, with online guides, that can be implemented in many LMICs. China and India have been using such methods to manage many conditions including depression, anxiety, other mental health challenges ^85^. Introducing these interventions among university students will most likely require a culturally acceptable method to handle burnout. The role of the university in these interventions starts with education around the importance of the intervention to all their students coupled with providing coaches to guide students to perfect these self-administered interventions.

**Strengths of the study**

The literature search was done on several databases including articles in multiple languages. The studies all used standardized tools for measuring burnout. The study has a sub-analysis of burnout from different country income status, a previously not explored area by other meta-analyses.

**Limitations**

This review had several limitations. The authors included studies from both medical and non-medical students. They may not be comparable in the levels of burnout experienced. The study included postgraduate and undergraduate students who may have differing levels of stress. The different scoring methods used to determine burnout were not considered in this study. There was still marked heterogeneity of the results mainly among countries from different economic statuses.

**Future direction**

We suggest the following to increase the understanding of burnout in LMICs. Longitudinal studies to identify the impact of education on student burnout. More studies are needed in non-medical students as they have been neglected in most of the studies. There is also a need to validate more tools in the different populations of LMICs to enable more reliable studies to be conducted in such an economic environment.

**Conclusion**

Burnout among university students in LMICs was low. The commonest screening tool used is the MBI and it showed lower burnout levels than other tools. Few studies have been conducted among university students especially in LICs and no observed variability in the use of other screening tools. This suggests the need for more studies to understand burnout and its associated factors in LMICs as well as validation of various freely available tools for use in these countries.

**Funding:** None

**Declaration of Competing Interest:** No conflict of interest for any author

**Acknowledgment**

Liberian Wilson Adriko who assisted with the literature search, The African Centre for Suicide Prevention and Research which provided training on the conduction of systematic review and meta-analysis.

**References**

1.	Kilbourne AM, Beck K, Spaeth‐Rublee B, et al. Measuring and improving the quality of mental health care: a global perspective. 2018;17(1):30-38.

2.	WHO WHO. Burn-out an “occupational phenomenon”. 2019; <http://www.who.int/mental_health/evidence/burn-out/en/>.

3.	Costa EFdO, Santos SA, Santos ATRdA, Melo EVd, Andrade TMdJC. Burnout Syndrome and associated factors among medical students: a cross-sectional study. 2012;67(6):573-580.

4.	Galbraith CS, Merrill GBJJof, Education H. Academic performance and burnout: An efficient frontier analysis of resource use efficiency among employed university students. 2015;39(2):255-277.

5.	Wing T, Pey YC, Subramaniam V, et al. Prevalence of Burnout in Medical and Non-medical Undergraduate Malaysian Students in Various International Universities-A Cross-Sectional Study. 2018:1-13.

6.	Rodriguez SYS, Carlotto MSJEdp. Predictors of Burnout Syndrome in psychologists. 2017;34(1):141-150.

7.	Dyrbye L, Shanafelt TJMe. A narrative review on burnout experienced by medical students and residents. 2016;50(1):132-149.

8.	Pharasi S, Patra SJIJoP. Burnout in medical students of a tertiary care Indian medical center: How much protection does resilience confer? 2020;62(4):407.

9.	Merces MCd, Coelho JMF, Lua I, et al. Burnout syndrome and metabolic syndrome: a cross-sectional population-based study. 2020:1-9.

10.	Alsaad A, Alkhalaf AB, Alali KM, Alibrahim JH, Almahdi ISJMS. Burnout prevalence and associated risk factors among KFU students, Alhasa, Saudi Arabia. 2021;25(107):146-156.

11.	Kajjimu J, Kaggwa MM, Bongomin F. Burnout and Associated Factors Among Medical Students in a Public University in Uganda: A Cross-Sectional Study. 2021; 12:63.

12.	Fares J, Al Tabosh H, Saadeddin Z, El Mouhayyar C, Aridi HJNAjoms. Stress, burnout and coping strategies in preclinical medical students. 2016;8(2):75.

13.	IsHak WW, Lederer S, Mandili C, et al. Burnout during residency training: a literature review. 2009;1(2):236.

14.	Goodger K, Gorely T, Lavallee D, Harwood CJTsp. Burnout in sport: A systematic review. 2007;21(2):127-151.

15.	Rodrigues H, Cobucci R, Oliveira A, et al. Burnout syndrome among medical residents: A systematic review and meta-analysis. 2018;13(11):e0206840.

16.	Frajerman A, Morvan Y, Krebs M-O, Gorwood P, Chaumette BJEP. Burnout in medical students before residency: a systematic review and meta-analysis. 2019; 55:36-42.

17.	Chunming WM, Harrison R, MacIntyre R, Travaglia J, Balasooriya CJBme. Burnout in medical students: a systematic review of experiences in Chinese medical schools. 2017;17(1):1-11.

18.	Stroup DF, Berlin JA, Morton SC, et al. Meta-analysis of observational studies in epidemiology: a proposal for reporting. 2000;283(15):2008-2012.

19.	Moher D, Liberati A, Tetzlaff J, Altman DG, medicine PGJP. Preferred reporting items for systematic reviews and meta-analyses: the PRISMA statement. 2009;6(7): e1000097.

20.	The World Bank W. World Bank Country and Lending Groups. 2021; <https://datahelpdesk.worldbank.org/knowledgebase/articles/906519-world-bank-country-and-lending-groups>. Accessed February 1, 2021, 2021.

21.	JBI JBI. The Joanna Briggs Institute Critical Appraisal tools for use in JBI Systematic Reviews. Checklist for Prevalence Studies. 2017; <https://jbi.global/sites/default/files/2019-05/JBI_Critical_Appraisal-Checklist_for_Prevalence_Studies2017_0.pdf>. Accessed 03/01/2021, 2021.

22.	Ma X, Lin L, Qu Z, Zhu M, Chu HJE. Performance of between-study heterogeneity measures in the Cochrane library. 2018;29(6):821.

23.	Franco GP, Barros A, Nogueira-Martins LA, Zeitoun SSJREEU. Burnout in nursing residents. 2011;45(1):12-18.

24.	Waldman SV, Diez JCL, Arazi HC, Linetzky B, Guinjoan S, Grancelli HJAP. Burnout, perceived stress, and depression among cardiology residents in Argentina. 2009;33(4):296-301.

25.	Jovanović N, Podlesek A, Volpe U, et al. Burnout syndrome among psychiatric trainees in 22 countries: Risk increased by long working hours, lack of supervision, and psychiatry not being first career choice. 2016; 32:34-41.

26.	Martins AE, Davenport MC, Del Valle MdlP, et al. Impact of a brief intervention on the burnout levels of pediatric residents. 2011;87(6):493-498.

27.	Nikodijević A, Labrović JA, Đoković AJJfT, Management P. Academic burnout among students at faculty of organizational sciences. 2012; 64:64-53.

28.	Tavares KFA, Souza N, Silva LDd, Kestenberg CCFJAPE. Prevalence of burnout syndrome among resident nurses. 2014;27(3):260-265.

29.	Mason HD, Nel JAJJoPiA. Compassion fatigue, burnout and compassion satisfaction: Prevalence among nursing students. 2012;22(3):451-455.

30.	Mafla A, Villa‐Torres L, Polychronopoulou A, et al. Burnout prevalence and correlates amongst Colombian dental students: the STRESSCODE study. 2015;19(4):242-250.

31.	das Neves CP, Ribeiro DMJRdA. Burnout em estudantes de graduação em Odontologia. 2016;16(1):39-49.

32.	Talih F, Warakian R, Ajaltouni J, Tamim HJAP. Correlates of depression and burnout among residents in a lebanese academic medical center: a cross-sectional study. 2016;40(1):38-45.

33.	Bera T, Mandal A, Bhattacharya S, Biswas NM, Ghosh A, Bera SJIMG. Burnout among medical students—a study across three medical colleges in Eastern India. 2013; 2013:356-359.

34.	Almeida GdC, Souza HRd, Almeida PCd, Almeida BdC, Almeida GHJAoCP. The prevalence of burnout syndrome in medical students. 2016;43(1):6-10.

35.	Osorio LP, Castaño LR, Córdoba IP, Ausecha GC, Aguirre AVJRCdSO. Correlación en Síndrome de Burnout y estrés académico en estudiantes de medicina de una facultad de salud en Cali, Colombia. 2015;5(3):31-34.

36.	Pereira-Lima K, Loureiro SRJEdP. Associations between social skills and burnout dimensions in medical residents. 2017;34(2):281-292.

37.	Galdino MJQ, Martins JT, Haddad MdCFL, Robazzi MLdCC, Birolim MMJAPdE. Síndrome de Burnout entre mestrandos e doutorandos em enfermagem. 2016;29(1):100-106.

38.	Tian L, Pu J, Liu Y, et al. Relationship between burnout and career choice regret among Chinese neurology postgraduates. 2019;19(1):1-10.

39.	Zhou W, Pu J, Zhong X, et al. Overlap of burnout-depression symptoms among Chinese neurology graduate students in a national cross-sectional study. 2021;21(1):1-9.

40.	Stein C, Sibanda TJAJoHPE. Burnout among paramedic students at a university in Johannesburg, South Africa. 2016;8(2):193-195.

41.	Barbosa ML, Ferreira BLR, Vargas TN, et al. Burnout prevalence and associated factors among Brazilian medical students. 2018; 14:188.

42.	Wickramasinghe ND, Dissanayake DS, Abeywardena GSJC, psychiatry a, health m. Prevalence and correlates of burnout among collegiate cycle students in Sri Lanka: a school-based cross-sectional study. 2018;12(1):1-11.

43.	Fontana MCP, Generoso IP, Sizilio A, Bivanco-Lima DJBme. Burnout syndrome, extracurricular activities and social support among Brazilian internship medical students: a cross-sectional analysis. 2020;20(1):1-9.

44.	Liu H, Yansane AI, Zhang Y, Fu H, Hong N, Kalenderian EJM. Burnout and study engagement among medical students at Sun Yat-sen University, China: A cross-sectional study. 2018;97(15).

45.	Malik AA, Bhatti S, Shafiq A, et al. Burnout among surgical residents in a lower-middle income country–Are we any different? 2016; 9:28-32.

46.	Mathias CT, Wentzel DLJC. Descriptive study of burnout, compassion fatigue and compassion satisfaction in undergraduate nursing students at a tertiary education institution in KwaZulu-Natal. 2017;40(1):1-6.

47.	Atlam SAJEJoCM. Burnout Syndrome: Determinants and Predictors among Medical Students of Tanta University, Egypt. 2018;36(1).

48.	Serrano FT, Salguero-Sánchez J, Ayala-Fernández J, García-Torres MF, Meza JC, Mejía CJC. Síndrome de Burnout en estudiantes de seis facultades de medicina de Colombia, 2016-1: estudio multicéntrico. 2016;21(2):29-34.

49.	Lee K, Yeung N, Wong C, Yip B, Luk LH, Wong SJPo. Prevalence of medical students’ burnout and its associated demographics and lifestyle factors in Hong Kong. 2020;15(7): e0235154.

50.	Calcides DAP, Didou RdN, Melo EVd, Oliva-Costa EFdJRdAMB. Burnout Syndrome in medical internship students and its prevention with Balint Group. 2019;65(11):1362-1367.

51.	Haile YG, Senkute AL, Alemu BT, Bedane DM, Kebede KBJBme. Prevalence and associated factors of burnout among Debre Berhan University medical students: a cross-sectional study. 2019;19(1):1-11.

52.	Lopes AR, Nihei OKJRbde. Burnout among nursing students: predictors and association with empathy and self-efficacy. 2020;73(1).

53.	Tlili MA, Aouicha W, Sahli J, et al. Prevalence of burnout among health sciences students and determination of its associated factors. 2021;26(2):212-220.

54.	Vidhukumar K, Hamza MJIjopm. Prevalence and Correlates of Burnout among Undergraduate Medical Students-A Cross-sectional Survey. 2020;42(2):122-127.

55.	Vasconcelos EMd, Trindade CO, Barbosa LR, Martino MMFdJRdEdEdU. Predictive factors of burnout syndrome in nursing students at a public university. 2020;54.

56.	Boni RAdS, Paiva CE, De Oliveira MA, Lucchetti G, Fregnani JHTG, Paiva BSRJPo. Burnout among medical students during the first years of undergraduate school: Prevalence and associated factors. 2018;13(3): e0191746.

57.	Muller M-E, Rusca S, Lazor-Blanchet CJRms. Burnout among physicians: a new related reason? 2020;16(709):1881-1885.

58.	Alhaffar BA, Abbas G, Alhaffar AAJJoOM, Toxicology. The prevalence of burnout syndrome among resident physicians in Syria. 2019;14(1):1-8.

59.	Tavares HHF, da Silva HRS, Miranda IMM, Braga MS, de Oliveira Santos R, Guerra HS. Factors associated with Burnout Syndrome in medical students.

60.	Ji L, Xiaowei Z, Ling K, Yao F, Qingkun S, Jun ZJM. Burnout level and job satisfaction in Chinese pediatrics residents: a web-based cross-sectional study. 2020;99(8).

61.	Bolatov AK, Seisembekov TZ, Askarova AZ, Igenbayeva BB, Smailova DS, Hosseini H. Burnout syndrome among medical students in Kazakhstan. 2021.

62.	Daud N, Pa MNM, Abdul AF, Rahim AA, Hassan NMJs. ACADEMIC FACTORs AssOCIATED WITh BuRNOuT IN MALAYsIAN MEDICAL sTuDENTs: A CROss-sECTIONAL sTuDY.5:7.

63.	MAGRI LV, MELCHIOR MdO, JARINA L, SIMONAGGIO FF, BATAGLION CJR-RGdO. Temporomandibular disorders and Burnout syndrome in students of the eighth period of dentistry course. 2019;67.

64.	Pokhrel NB, Khadayat R, Tulachan PJBp. Depression, anxiety, and burnout among medical students and residents of a medical school in Nepal: a cross-sectional study. 2020;20(1):1-18.

65.	Aghagari Z, Ahmad M, Borhani FJQUoMSJ. Prevalence of Academic Burnout and Its Related Factors among Nursing Student in Tehran Shahid Beheshti University of Medical Sciences, 2015, (Iran). 2019;13(8):50-61.

66.	Geng H, Tan F, Deng Y, et al. High rate of burnout among residents under standardized residency training in a tertiary teaching hospital of middle China: Results from a cross-sectional survey. 2020;99(27).

67.	Khosravi MJEJoTM. Burnout among Iranian medical students: Prevalence and its relationship to personality dimensions and physical activity. 2021.

68.	Ogboghodo EO, Edema OMJNPMJ. Assessment of burnout amongst resident doctors in Benin City, Edo State, Nigeria. 2020;27(3):215.

69.	Zhang J-Y, Shu T, Xiang M, Feng Z-CJFiP. Learning Burnout: Evaluating the Role of Social Support in Medical Students. 2021; 12:277.

70.	Ogoma SOJJoP. Problem-focused Coping Controls Burnout in Medical Students: The Case of a Selected Medical School in Kenya. 2020;8(1):69-79.

71.	Verougstraete D, Hachimi Idrissi SJACB. The impact of burn-out on emergency physicians and emergency medicine residents: a systematic review. 2020;75(1):57-79.

72.	Salvagioni DAJ, Melanda FN, Mesas AE, González AD, Gabani FL, Andrade SMdJPo. Physical, psychological and occupational consequences of job burnout: A systematic review of prospective studies. 2017;12(10): e0185781.

73.	Rybarczyk MM, Schafer JM, Elm CM, et al. Prevention of burn injuries in low-and middle-income countries: a systematic review. 2016;42(6):1183-1192.

74.	Lucchetti G, Damiano RF, DiLalla LF, et al. Cross-cultural differences in mental health, quality of life, empathy, and burnout between US and Brazilian medical students. 2018;42(1):62-67.

75.	Tang L, Zhang F, Yin R, Fan ZJFiP. Effect of Interventions on Learning Burnout: A Systematic Review and Meta-Analysis. 2021; 12:533.

76.	Wang Y, Xiao H, Zhang X, Wang LJFiP. The role of active coping in the relationship between learning burnout and sleep quality among college students in China. 2020;11.

77.	Kajjimu J, Kaggwa MM, Bongomin F. Response to the Letter to the Editor Regarding Our Feature "Burnout and Associated Factors Among Medical Students in a Public University in Uganda: A Cross-Sectional Study" [Response to Letter]. *Adv Med Educ Pract.* 2021; 12:281-283.

78.	Shoman Y, Marca S, Bianchi R, et al. Psychometric properties of burnout measures: a systematic review. 2021;30.

79.	Dugani S, Afari H, Hirschhorn LR, et al. Prevalence and factors associated with burnout among frontline primary health care providers in low-and middle-income countries: a systematic review. 2018;2.

80.	NAS NAoS. Valid and Reliable Survey Instruments to Measure Burnout, Well-Being, and Other Work-Related Dimensions. 2021; <https://nam.edu/valid-reliable-survey-instruments-measure-burnout-well-work-related-dimensions/>. Accessed 03/03/2021, 2021.

81.	West CP, Dyrbye LN, Erwin PJ, Shanafelt TDJTL. Interventions to prevent and reduce physician burnout: a systematic review and meta-analysis. 2016;388(10057):2272-2281.

82.	Goodman MJ, Schorling JBJTIJoPiM. A mindfulness course decreases burnout and improves well-being among healthcare providers. 2012;43(2):119-128.

83.	Ofei-Dodoo S, Cleland-Leighton A, Nilsen K, Cloward JL, Casey EJJoo, medicine e. Impact of a mindfulness-based, workplace group yoga intervention on burnout, self-care, and compassion in health care professionals: a pilot study. 2020;62(8):581-587.

84.	Kinnunen SM, Puolakanaho A, Mäkikangas A, Tolvanen A, Lappalainen RJIJoSM. Does a mindfulness-, acceptance-, and value-based intervention for burnout have long-term effects on different levels of subjective well-being? 2020;27(1):82.

85.	Ansley BM, Houchins DE, Varjas K, et al. The impact of an online stress intervention on burnout and teacher efficacy. 2021; 98:103251.

86.	Rodrigues, C. S., Deus, M. L. A. D., Andrade, F. T. D., Rezende, G. B., Mariano, L. D. Á., & Sé, A. B. (2020). Evaluation of Burnout Syndrome Prevalence in Medical Students. *Revista Brasileira de Educação Médica*, *44*(4).

**Figure 1: The PRISMA flow diagram**

**Figure 2: Forest plot of the prevalence of burnout in LMICs**

**Figure 3: Forest plot of the prevalence of emotional exhaustion in LMICs**

**Figure 4: Forest plot of the prevalence of cynicism in LMICs**

**Figure 5: Forest plot of reduced professional efficacy**

**Supplement file 1: Search strategy**

**Supplementary file 2: Quality assessment of the included studies**

**Supplementary file 3: The funnel plot for burnout prevalence**

**Supplementary file 4: The funnel plot of the prevalence of emotional exhaustion in LMICs**

**Supplementary file 5: Funnel plot of the prevalence of cynicism in LMICs**

**Supplementary file 6. Funnel plot of the prevalence of professional efficacy in LMICs**

**Supplementary file 7: Forest plot for the burnout measurement tools**

**Supplementary file 8: PRISMA Checklist**
